# Study of Anatomy in Egypt

**Published:** 1852-01

**Authors:** 


*Study of Anatomy in Egypt.*—At the time when Mahomet Ali was persuaded by Clot Bey to organize a school of medicine in Cairo, the old man consulted the Council of the Ulama, or learned men of the orthodox sects, in regard to the legality of dissecting human bodies, for the purpose of anatomical knowledge. They decided that it would be repugnant to religion. However, he said nothing, and gave directions to his surgeon to proceed. He had the united force of Musselman prejudices and Moslem bigotry marshalled against him; but he was the pillar that sustained the entire fabric of Egyptian government, against whose orders no one dared raise the voice of remonstrance, however much they might growl over an infraction of the Koran. He lived long enough to have his bigoted subjects become familiar with dissections, and till the four orthodox sects of the Hanafees, Shafees, Málikees and Hambelees, had lost their influence over the ignorant rabble of the metropolis. Anatomical pursuits are now conducted there on a proper scale, and under fewer restrictions than in several of these United States, where a physician and surgeon are required to understand anatomy in order to practice, and yet they are liable to prosecution, to fine and imprisonment, for doing precisely what they are required to do. Mahomet Ali was in advance of the age in his dominions, and far wiser and more liberal than some of the enlightened Christian legislators of these United States.—*Bos. Med. and Surg. Journal.*

